# Der Zusammenhang zwischen Adipositas, sozialer Isolation und psychischer Gesundheit – Ergebnisse der LIFE-Adult-Studie

**DOI:** 10.1007/s00103-024-03940-3

**Published:** 2024-08-27

**Authors:** Charlyn Görres, Jana Hoßbach, Alexander Pabst, Melanie Luppa, Janine Stein, Franziska D. Welzel, Franziska U. Jung, Felix S. Hussenoeder, Christoph Engel, Toralf Kirsten, Nigar Reyes, Kerstin Wirkner, Steffi G. Riedel-Heller, Margrit Löbner

**Affiliations:** 1 Institut für Sozialmedizin, Arbeitsmedizin und Public Health (ISAP), Medizinische Fakultät, Universität Leipzig, Leipzig, Deutschland; 2 Institut für Medizinische Informatik, Statistik und Epidemiologie, Universität Leipzig, Leipzig, Deutschland; 3 Medizininformatikzentrum – Abteilung Medical Data Science, Universitätsklinikum Leipzig AöR, Leipzig, Deutschland; 4 LIFE – Leipziger Forschungszentrum für Zivilisationserkrankungen, Universität Leipzig, Leipzig, Deutschland

**Keywords:** Adipositas, Depressivität, Psychische Gesundheit, Soziale Isolation, Prävalenz, Obesity, Depressive symptoms, Mental health, Social isolation, Prevalence

## Abstract

**Hintergrund:**

Bevölkerungsbasierte Studien zum Zusammenhang von sozialer Isolation und Adipositas, die auch jüngere Erwachsene einschließen, fehlen in Deutschland bisher. Ziel der vorliegenden Arbeit ist die Untersuchung der Prävalenzen sozialer Isolation bei Menschen mit und ohne Adipositas. Zudem werden sozial Isolierte mit und ohne Adipositas hinsichtlich soziodemografischer und sozioökonomischer Faktoren sowie depressiver Symptomatik untersucht.

**Methoden:**

Grundlage waren die Baseline-Daten der LIFE-Adult-Studie (18–79 Jahre) aus dem Studienzeitraum 2011–2014. Die untersuchte Stichprobe umfasste *n* = 8350 Teilnehmende. Erhoben wurden neben soziodemografischen Charakteristika und dem sozioökonomischen Status (SES), Daten zur sozialen Isolation (LSNS-6), zu Depressivität (ADS) und Body-Mass-Index (BMI). Die Auswertungen erfolgten mittels inferenzstatistischer Analysen und linearer Regression.

**Ergebnisse:**

Insgesamt waren 13,1 % der Gesamtstichprobe von sozialer Isolation betroffen. Teilnehmende mit Adipositas (20,4 %) wiesen eine signifikant (*p* < 0,001) höhere Prävalenz als jene ohne Adipositas auf (11,4 %). Eine bessere soziale Einbindung war signifikant mit jüngerem Alter (*p* < 0,001), weiblichem Geschlecht (*p* < 0,001), einem verheirateten (und zusammenlebenden) Familienstand (*p* < 0,001), einem höheren sozioökonomischen Status (*p* < 0,001) sowie einer geringeren depressiven Symptomatik (*p* < 0,001) assoziiert.

**Diskussion:**

Ein höherer BMI ging nicht per se mit einer schlechteren sozialen Einbindung einher. Es zeigte sich jedoch, dass sozial isolierte Menschen mit Adipositas im Vergleich zu jenen ohne Adipositas eine besondere Risikogruppe für eine eingeschränkte psychische Gesundheit darstellen und eine doppelt so hohe Prävalenz von sozialer Isolation aufwiesen.

## Abstract (en)

**Background:**

Population-based studies on the relationship between social isolation and obesity, which also include younger adults, are still lacking in Germany. The aim of this study is to investigate the prevalence of social isolation in people with and without obesity. In addition, socially isolated people with and without obesity are examined with regard to socio-demographic and socio-economic factors as well as with regard to depressive symptoms.

**Methods:**

This study was based on baseline data from the LIFE Adult Study (18–79 years) from the 2011–2014 study period. The sample comprised *n* = 8350 participants. In addition to socio-demographic characteristics and socio-economic status (SES), data on social isolation (LSNS-6), depression (ADS), and body mass index (BMI) were collected. Evaluations were carried out using inferential statistical analyses.

**Results:**

Overall, 13.1% of the total sample were affected by social isolation. Participants with obesity (20.4%) had a significantly (*p* < 0.001) higher prevalence compared to those without obesity (11.4%). A better social integration was significantly associated with younger age (*p* < 0.001), female sex (*p* < 0.001), being married (and cohabiting) (*p* < 0.001), higher socioeconomic status (*p* < 0.001), and lower depressive symptoms (*p* < 0.001).

**Discussion:**

A higher BMI was not associated per se with poorer social integration. However, the present study showed that socially isolated people with obesity represent a special risk group for impaired mental health and had twice the prevalence of social isolation compared to those without obesity.

## Hintergrund

Soziale Isolation ist mit weitreichenden gesundheitlichen Konsequenzen verbunden [1]. So gilt mittlerweile als unbestritten, dass soziale Isolation das Risiko für körperliche Erkrankungen erhöht [2]. Soziale Isolation macht jedoch nicht nur krank, sie erhöht auch maßgeblich das Mortalitätsrisiko [1, 3]. Vor allem in den westlichen Industrieländern rücken im Zuge des 21. Jahrhunderts fortlaufend Debatten über eine Zunahme der sozialen Isolation oder eine „Einsamkeitsepidemie“ in den Vordergrund [4]. Die Identifikation möglicher Risikogruppen für das Erleben sozialer Isolation erweist sich in Anbetracht der immensen Folgen als zentrale Aufgabe der Public-Health-Forschung.

Gleichzeitig stellen Menschen mit Adipositas eine wachsende Bevölkerungsgruppe dar. Den Ergebnissen der Studie zur Gesundheit Erwachsener (erste Erhebungswelle des Deutschen Erwachsenen Gesundheitssurveys [DEGS1] 2008–2011) zufolge gilt dabei knapp ein Viertel der deutschen Erwachsenen als adipös, wobei Männer und Frauen gleichermaßen betroffen sind (23,3 % Männer, 23,9 % Frauen; [5]). Adipositas und ihre Begleiterkrankungen sind mit physischen Einschränkungen und erheblichen psychischen und psychosozialen Belastungen verbunden, die sich negativ auf die psychosoziale Funktionsfähigkeit auswirken [6]. So geht die Erkrankung beispielsweise mit Einschränkungen im Bewegungsapparat und damit verbundenen Beeinträchtigungen im Alltag einher [7, 8].

Darüber hinaus sind Menschen mit Adipositas einer gewichtsbezogenen Stigmatisierung und Diskriminierung ausgesetzt [9, 10]. Eine aufgrund des Gewichts eingeschränkte Mobilität kann insbesondere bei schweren Adipositasformen die Aufrechterhaltung sozialer Kontakte erschweren [11]. Ein Gefühl der Stigmatisierung und Ablehnung sowie Diskriminierungserfahrungen können die sozialen Interaktionen beeinträchtigen bzw. eine soziale Isolation fördern [12, 13].

Wie vorherige Forschung bereits zeigen konnte, steht soziale Isolation auch im Zusammenhang mit Depressivität [14–16]. Beispielsweise gilt neben Adipositas als körperlicher Risikofaktor auch soziale Isolation als psychosozialer Risikofaktor für eine unipolare Depression [17, 18]. Auch ein reziproker Zusammenhang, bei welchem eine Depression das Erleben sozialer Isolation begünstigt, wurde in der Forschungsliteratur bereits diskutiert [19]. Gleichermaßen treten Adipositas und Depression häufig komorbid auf [20]. Verschiedene Studien zeigen einen wechselseitigen Zusammenhang der Erkrankungen auf [21, 22]. Von Adipositas betroffene Personen mit einer komorbiden depressiven Störung stellen für das klinische Versorgungssystem eine besondere Herausforderung dar [20]. Bisherige Studien zum Zusammenhang von sozialer Isolation/Einsamkeit und Adipositas lieferten heterogene Evidenz, bezogen sich dabei jedoch nahezu ausschließlich auf Personen mittleren Alters oder ältere und hochaltrige Bevölkerungsschichten. Bevölkerungsbasierte Studien zum Zusammenhang von sozialer Isolation und Adipositas, die gleichermaßen jüngere und ältere Erwachsene einbeziehen und zur Aufklärung heterogener Studienergebnisse beitragen können, fehlen indessen. So liegen bisher auch keine Studien über die Prävalenz komorbider Erscheinungsformen von Adipositas und sozialer Isolation in der deutschen Allgemeinbevölkerung vor.

Vor dem Hintergrund hoher Risiken sowohl für die mentale und körperliche Gesundheit bei sozialer Isolation als auch Adipositas stellen sich daher folgende Fragen, welche hier im Rahmen einer bevölkerungsbasierten Kohortenstudie (18–79 Jahre) untersucht werden sollen:

1. Wie hoch ist die Prävalenz von sozialer Isolation bei Menschen mit Adipositas im Vergleich zu Menschen ohne Adipositas?
2. Unterscheiden sich sozial Isolierte mit Adipositas von sozial Isolierten ohne Adipositas in Bezug auf soziodemografische und sozioökonomische Faktoren sowie in Bezug auf die psychische Gesundheit (Depression)?
3. Welche soziodemografischen, sozioökonomischen und gesundheitsbezogenen Faktoren sind mit einer besseren sozialen Einbindung assoziiert?

## Methoden

### Stichprobe

**Abb. 1:**
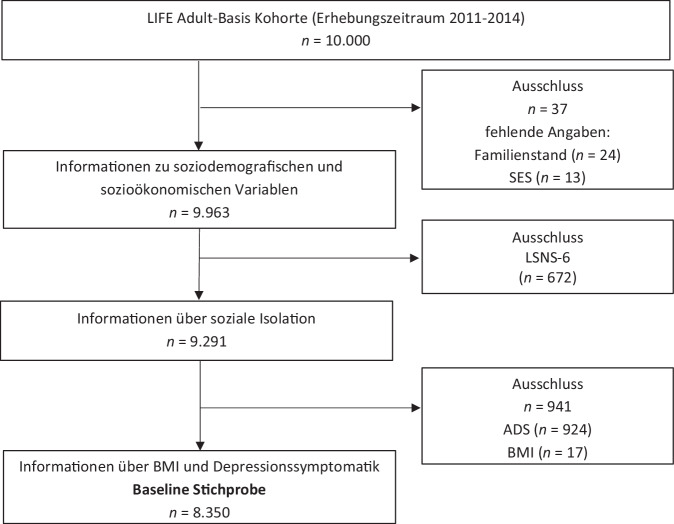
Selektionsprozess der Studienstichprobe. Flussdiagramm. *ADS* Allgemeine Depressionsskala, *LSNS‑6* Kurzversion der Lubben Social Network Scale, *BMI* Body-Mass-Index, *SES* sozioökonomischer Status. Quelle: eigene Abbildung

In der vorliegenden Studie wurden Querschnittsdaten der Baseline-Erhebung der LIFE-Adult-Studie im Rahmen einer Sekundäranalyse ausgewertet. Detaillierte Angaben zum Studienprozedere wurden bereits publiziert [23, 24]. Bei der LIFE-Adult-Studie handelt es sich um eine vom Leipziger Forschungszentrum für Zivilisationskrankheiten (LIFE) durchgeführte längsschnittlich angelegte bevölkerungsbasierte Kohortenstudie, welche die Gesundheit der Bevölkerung Leipzigs abbildet und Risikofaktoren für ausgewählte Zivilisationskrankheiten erforscht. Für den Baseline-Zeitpunkt lagen Daten von *n* = 10.000 Teilnehmenden im Alter von 18–79 Jahren vor. Diese wurden im Zeitraum von August 2011 bis Dezember 2014 als ersten Erhebungszeitpunkt einer Längsschnittstudie erhoben. Wie in Abb. 1 dargestellt, wurden sukzessiv Proband:innen mit fehlenden Werten ausgeschlossen. In einem ersten Schritt wurden Teilnehmende aufgrund fehlender soziodemografischer und sozioökonomischer Informationen ausgeschlossen (*n* = 37). Im zweiten Schritt wurden *n* = 672 Personen aufgrund fehlender Daten hinsichtlich der sozialen Isolation exkludiert. Die verbleibende Kohorte wurde um *n* = 941 Personen aufgrund fehlender Werte in der Depressionsskala und zum BMI reduziert. Die Analysestichprobe der vorliegenden Studie belief sich damit auf *n* = 8350 Teilnehmende.

### Datenerhebung

#### Soziale Isolation

Die soziale Isolation wurde mithilfe der Kurzversion der Lubben Social Network Scale (LSNS‑6; [25]), ein in der Forschung weit etabliertes Selbstbeurteilungsinstrument, erfasst. Es handelt sich um ein quantitatives Maß für die soziale Netzwerkgröße mit dem Ziel, soziale Isolation anhand der Anzahl, Häufigkeit und Nähe der Sozialkontakte mit Familienangehörigen, Freunden und Nachbarn unter Berücksichtigung der von ihnen erhaltenen sozialen Unterstützung zu messen [25]. In Anlehnung an Gironda und Lubben (2003) soll soziale Isolation in der vorliegenden Arbeit daher als ein unzureichendes soziales Netzwerk, aus dem eine Person schöpfen oder auch soziale Unterstützung austauschen kann, definiert sein [26]. Mithilfe von insgesamt 6 Items werden Informationen zur Anzahl, Häufigkeit und Nähe der Kontakte mit familiär und freundschaftlich verbundenen Bezugspersonen erfasst, die für soziale Unterstützung/Hilfe und private Gespräche zur Verfügung stehen. Die Antworten erfolgen auf einer 5‑stufigen Skala mit den Optionen 0 = „keine“, 1 = „1“, 2 = „2“, 3 = „3 oder 4“, 4 = „5 bis 8“ und 5 = „9 oder mehr“, die jeweils die Anzahl der Familienangehörigen bzw. Freunde/Nachbarn angeben. Der resultierende Gesamtsummenscore reicht von 0 bis 30, wobei ein höherer Wert ein größeres soziales Netzwerk indiziert. Zur Operationalisierung sozialer Isolation wurde der LSNS-6-Summenscore anhand des Cut-off-Wertes von 12 dichotomisiert in „sozial nicht isoliert/nein“ (LSNS-6 ≥ 12) und „sozial isoliert/ja“ (LSNS-6 < 12; [25]). Höhere Werte des Summenscores können im Sinne einer besseren sozialen Einbindung interpretiert werden.

#### Adipositas

Zur Klassifikation von Adipositas wurde der Body-Mass-Index (BMI) als Kriterium herangezogen. Seine Berechnung erfolgte der Weltgesundheitsorganisation (WHO) entsprechend anhand des Verhältnisses von Körpergewicht zum Quadrat der Körpergröße (kg/m^2^; [27]). Körpergröße und -gewicht wurden zum Baseline-Zeitpunkt nach standardisiertem Verfahren durch geschulte Study Nurses objektiv gemessen. Zur Differenzierung von Menschen mit und ohne eine zur Baseline bestehende Adipositas wurde eine dichotome Variable (BMI <30 kg/m^2^: „keine Adipositas“/BMI ≥30 kg/m^2^: „Adipositas“) gebildet.

#### Depressive Symptome

Zur Erfassung einer depressiven Symptomatik fand die Allgemeine Depressionsskala (ADS) Anwendung, die deutsche Version der Center for Epidemiologic Studies Depression Scale (CES‑D; [28, 29]). Bei der ADS handelt es sich um ein weitverbreitetes und zur Messung depressiver Symptome in der Allgemeinbevölkerung bewährtes Selbstbeurteilungsinstrument, welches vielfach validiert wurde. Mittels 20 Items kann auf einer 4‑stufigen Antwortskala mit den Optionen „selten, nie“, „manchmal“, „öfter“ und „meistens“ die Häufigkeit des Auftretens depressiver Symptome während der letzten Woche beurteilt werden. Die Auswertung der Skala erfolgt durch Addition der einzelnen Punktwerte (0–3 Punkte) zu einem Summenscore zwischen 0 und 60, wobei ein höherer Wert für eine stärker ausgeprägte depressive Symptomatik steht. Um das Vorliegen einer depressiven Symptomatik bestimmen zu können, wurde der empfohlene Cut-off-Wert von ≥23 genutzt [29, 30].

#### Soziodemografische und sozioökonomische Angaben

Soziodemografische Informationen umfassten das Alter, das Geschlecht sowie den Familienstand. Zudem wurden Angaben zum sozioökonomischen Status (SES) erfasst, eingeteilt nach etablierten Kriterien in die 3 Statusgruppen „niedrig“ (SES ≤9,2), „mittel“ (SES >9,2–15,3) und „hoch“ (SES >15,3; [31]).

### Statistische Analysen

Die Prävalenz von sozialer Isolation bei Menschen mit Adipositas im Vergleich zu Menschen ohne Adipositas wurde in Prozent mit 95 %-Konfidenzintervallen (95 %-KI) bezogen auf die Gesamtheit aller Probanden mit verwertbaren Antworten berechnet. Gruppenunterschiede in der Prävalenz wurden mit dem Chi-Quadrat-Test überprüft. Unterschiede zwischen sozial Isolierten mit und ohne Adipositas in Bezug auf soziodemografische und sozioökonomische Faktoren sowie in Bezug auf die psychische Gesundheit (Depression) wurden mittels Chi-Quadrat-Tests und unabhängigen t‑Tests untersucht. Bei Verletzung der Varianzhomogenität wurde ein Welch-t-Test durchgeführt. Zudem wurde eine lineare Regression in Bezug auf assoziierte Faktoren (Geschlecht, Alter, Familienstand, BMI, SES, Depressionssummenwert) für den Grad der sozialen Einbindung (abhängige Variable) durchgeführt. Höhere Werte der abhängigen Variable (LSNS-6) können im Sinne einer stärkeren sozialen Einbindung interpretiert werden. Die Verarbeitung der Daten erfolgte mit der Statistiksoftware IBM Statistics SPSS 29 für Windows sowie mit Stata 16.0 SE [32, 33]. Es wurde ein Signifikanzniveau von α = 0,05 für alle Analysen festgelegt. Für die Analysen wurde ein Gewichtungsfaktor gemäß den Daten des Zensus 2011 angewandt, um die Unterschiede in den Stichprobenanteilen der LIFE-Studie der deutschen Bevölkerungsstruktur hinsichtlich Alter und Geschlecht anzupassen.

## Ergebnisse

### Stichprobencharakteristika

Die soziodemografischen und sozioökonomischen Angaben sowie die relevanten gesundheitsbezogenen Charakteristika für die Gesamtstichprobe sowie für Menschen mit und ohne eine zum Baseline-Zeitpunkt bestehende Adipositas sind in Tab. 1 aufgeführt.

**Tab. 1:** Stichprobencharakteristika der Baseline-Gesamtstichprobe, stratifiziert im Hinblick auf Personen ohne und mit Adipositas

	Gesamtstichprobe		Menschen ohne Adipositas		Menschen mit Adipositas		*p*-Wert^b^
*Gesamt, N (%)*	*8350*	*(100,0)*	*6329*	*(81,3)*	*2021*	*(18,7)*	*–*
**Soziodemografische Charakteristika**							
*Alter M, (SD)*	47,3	(15,8)	45,5	(15, 7)	54,7	(13,8)	**< 0,001**
*Geschlecht, n (%)*							0,224
Männlich	3922	(48,4)	2964	(48,1)	958	(49,8)	–
Weiblich	4428	(51,6)	3365	(51,9)	1061	(50,2)	–
*Familienstand, n (%)*							**< 0,001**
Verheiratet, zusammenlebend	4992	(45,1)	3706	(42,5)	1286	(56,2)	–
Verheiratet, getrennt lebend	213	(2,0)	168	(1,9)	45	(2,0)	–
Ledig	1539	(39,3)	1264	(42,9)	275	(23,6)	–
Geschieden	1152	(10,3)	881	(9,7)	271	(12,5)	–
Verwitwet	454	(3,4)	310	(2,9)	144	(5,7)	–
**Sozioökonomische Charakteristika**							
*Sozioökonomischer Status, n (%)*							**< 0,001**
Niedrig	1544	(20,4)	1056	(19,0)	488	(26,3)	–
Mittel	5031	(59,3)	3833	(59,9)	1198	(56,7)	–
Hoch	1775	(20,3)	1440	(21,1)	335	(17,0)	–
**Gesundheitsbezogene Charakteristika**							
ADS-Score, *M, (SD)*	10,4	(7,0)	10,2	(6,9)	11,4	(7,0)	**< 0,001**
BMI, *M, (SD)*	26,2	(4,8)	24,4	(2,9)	33,9	(3,8)	**< 0,001**
LSNS-6-Score^a^, *M, (SD)*	17,4	(5,2)	17,6	(5,0)	16,4	(5,6)	**< 0,001**

Anmerkungen: fett markierte *p*-Werte sind signifikant

*M* Mittelwert, *SD* Standardabweichung, *%* gewichtet nach Alter und Geschlecht gemäß den Zensus-Daten 2011; *n* ungewichtete Zählungen, *ADS* Allgemeine Depressionsskala, *LSNS‑6* Kurzversion der Lubben Social Network Scale, *BMI* Body-Mass-Index

^a^Höhere Werte des LSNS-6-Scores zeigen eine höhere soziale Vernetzung an

^b^Ermittlung des *p*-Wertes für Gruppenunterschiede zwischen Menschen ohne und mit Adipositas anhand von Chi-Quadrat-Tests für kategoriale sowie von 2‑seitigen, unabhängigen t‑Tests für kontinuierliche Variablen; Anwendung der Korrektur nach Welch bei Verletzung der Varianzhomogenität

Das durchschnittliche Alter in der Gesamtstichprobe lag bei 47,3 Jahren (SD = 15,8) und 51,6 % der Teilnehmenden waren weiblich. Personen aus der Gesamtstichprobe lebten mehrheitlich verheiratet zusammen (45,1 %) oder waren ledig (39,3 %). Mit 59,3 % wies mehr als die Hälfte der Proband/-innen in der Gesamtstichprobe einen mittleren sozioökonomischen Status auf. In Bezug auf die gesundheitsbezogenen Faktoren zeigte sich in der Gesamtstichprobe ein mittlerer Depressionswert von 10,4 (SD = 7,0), ein durchschnittlicher BMI von 26,2 (SD = 7,0) sowie ein Mittelwert der sozialen Vernetzung von 17,4 (SD = 5,2). Statistisch bedeutsame Gruppenunterschiede zwischen Menschen mit und ohne Adipositas lagen hinsichtlich soziodemografischer und sozioökonomischer als auch bezüglich gesundheitsbezogener Indikatoren vor. Mit einem Durchschnittsalter von 54,7 Jahren (SD = 13,8) waren Menschen mit Adipositas signifikant älter als Menschen ohne Adipositas mit einem durchschnittlichen Alter von 45,5 Jahren (*p* < 0,001). Zudem unterschied sich der Familienstand zwischen den Gruppen signifikant (*p* < 0,001). So waren Menschen ohne Adipositas eher ledig (42,9 %) und Adipositas-Betroffene häufiger verheiratet, zusammenlebend (56,2 %). Auch das Vorliegen eines niedrigen, mittleren und hohen SES war in den Gruppen signifikant verschieden verteilt (*p* < 0,001). Fast ein Drittel der Menschen mit Adipositas (26,3 %) wies einen niedrigen SES auf. Im Vergleich dazu trat ein niedriger SES in der Stichprobe der Menschen ohne Adipositas mit 19,0 % weniger häufig auf. Menschen mit Adipositas zeigten zudem im Mittel eine signifikant höhere depressive Symptomatik (M = 11,4; SD = 7,0) als Menschen ohne Adipositas (M = 10,2; SD = 6,9; *p* < 0,001). Die soziale Vernetzung fiel mit einem durchschnittlichen LSNS-Score von 16,4 (SD = 5,6) bei Menschen mit Adipositas signifikant geringer aus als bei Menschen ohne Adipositas mit einem Durchschnittswert von 17,6 (SD = 5,0; *p* < 0,001).

### Prävalenz sozialer Isolation bei Menschen mit und ohne Adipositas

Tab. 2 zeigt die Prävalenz sozialer Isolation in der Gesamtstichprobe sowie im Vergleich zwischen Menschen mit und ohne Adipositas. Die Prävalenz für eine soziale Isolation betrug in der Gesamtstichprobe 13,1 %. Die Prävalenz sozialer Isolation lag bei Adipositas-Betroffenen mit 20,4 % signifikant höher als bei Personen ohne eine Adipositas mit einer Prävalenz von 11,4 % (*p* < 0,001). Dem Phi-Koeffizienten (φ = 0,104) zufolge handelt sich hierbei um einen schwachen Zusammenhang zwischen Adipositas und sozialer Isolation [34].

**Tab. 2:** Prävalenz sozialer Isolation bei Menschen ohne und mit Adipositas im Vergleich

Soziale Isolation	Gesamtstichprobe (*n* = 8350)			Menschen ohne Adipositas (*n* = 6329)			Menschen mit Adipositas (*n* = 2021)			*p*-Wert
	*n*	%	95 %-KI	*n*	%	95 %-KI	*n*	%	95 %-KI	
Ja	1391	13,1	[12,4; 13,7]	970	11,4	[10,6; 12,1]	421	20,4	[18,4; 22,4]	**< 0,001**
Nein	6959	87,0	[86,2; 87,7]	5359	88,6	[87,9; 89,4]	1600	79,7	[77,6; 81,6]	–

Anmerkungen: % und 95 %-KI sind gewichtet nach Alter und Geschlecht gemäß den Zensus-Daten 2011; Ermittlung des *p*-Wertes anhand eines (2-seitigen) Chi-Quadrat-Tests zur Bestimmung des Prävalenzunterschiedes sozialer Isolation zwischen Menschen ohne und mit Adipositas, fett markierte *p*-Werte sind signifikant

*n* ungewichtete Anzahl der Personen; *KI* Konfidenzintervall

### Gruppenvergleich sozial isolierter Menschen mit und ohne Adipositas

Tab. 3 bildet die Unterschiede zwischen den Gruppen sozial isolierter Personen mit und ohne Adipositas ab. Sozial isolierte Personen mit Adipositas (M = 55,9; SD = 13,3) waren durchschnittlich älter als Personen ohne Adipositas (M = 54,1; SD = 13,8; *p* = 0,019). Ebenso zeigte sich ein signifikanter Unterschied hinsichtlich der depressiven Symptomatik. So wiesen sozial isolierte Personen mit Adipositas im Mittel (M = 15,2; SD = 8,4) eine signifikant höhere depressive Symptomatik auf als sozial Isolierte ohne Adipositas (M = 13,9; SD = 8,4; *p* = 0,013). Es zeigten sich hingegen keine signifikanten Unterschiede hinsichtlich des Geschlechts, des Familienstandes oder in Bezug auf den SES.

**Tab. 3:** Vergleich sozial isolierter Personen ohne und mit Adipositas

	Sozial Isolierte ohne Adipositas (*n* = 970)				Sozial Isolierte mit Adipositas (*n* = 421)				
	*n*	(%)	*M*	*(SD)*	*n*	(%)	*M*	*(SD)*	*p*-Wert^a^
**Soziodemografische Charakteristika**									
*Alter*	–	–	54,1	(13,8)	–	–	55,9	(13,3)	**0,019**
*Geschlecht*	–	–	–	–	–	–	–	–	0,391
Männlich	539	(56,6)	–	–	217	(53,9)	–	–	–
Weiblich	431	(43,4)	–	–	204	(46,1)	–	–	–
*Familienstand*	–	–	–	–	–	–	–	–	0,631
Verheiratet, zusammenlebend	502	(43,4)	–	–	221	(46,7)	–	–	0,296
Verheiratet, getrennt lebend	34	(3,3)	–	–	13	(2,8)	–	–	0,645
Ledig	183	(30,4)	–	–	78	(25,9)	–	–	0,141
Geschieden	182	(17,3)	–	–	77	(18,4)	–	–	0,668
Verwitwet	69	(5,7)	–	–	31	(6,2)	–	–	0,774
**Sozioökonomische Charakteristika**									
*Sozioökonomischer Status*	–	–	–	–	–	–	–	–	0,252
Niedrig	277	(29,0)	–	–	149	(34,1)	–	–	0,102
Mittel	571	(58,5)	–	–	228	(55,0)	–	–	0,286
Hoch	122	(12,5)	–	–	44	(11,0)	–	–	0,491
**Psychische Gesundheit**									
*Depressive Symptomatik*^*b*^	–	–	13,9	(8,4)	–	–	15,2	(8,4)	**0,013**

Anmerkungen: fett markierte *p*-Werte sind signifikant

*M* Mittelwert, *SD* Standardabweichung, *%* gewichtet nach Alter und Geschlecht gemäß den Zensus-Daten 2011, *n* ungewichtete Zählungen

^a^Ermittlung des *p*-Wertes anhand von Chi-Quadrat-Tests für kategoriale sowie von einseitigen, unabhängigen t‑Tests für kontinuierliche Variablen; Anwendung der Korrektur nach Welch bei Verletzung der Varianzhomogenität

^b^Gemessen mit der Allgemeinen Depressionsskala (ADS*)*

### Soziale Einbindung und assoziierte Faktoren

Tab. 4 zeigt die Regressionsergebnisse hinsichtlich signifikanter Prädiktoren für die soziale Einbindung in der Gesamtstichprobe. Mit zunehmendem Alter reduzierte sich die soziale Einbindung signifikant (β = −0,11, *p* < 0,001). Weibliches Geschlecht war signifikant mit einer besseren sozialen Einbindung im Vergleich zu Männern assoziiert (β = 0,80, *p* < 0,001). Der Familienstand zeigte sich ebenfalls als signifikanter Prädiktor (F = 20,77, *p* < 0,001). Im Vergleich zu zusammenlebend Verheirateten hatten getrennt lebende Verheiratete (β = −1,40, *p* < 0,001), Ledige (β = −1,36, *p* < 0,001) und Geschiedene (β = −1,63, *p* < 0,001) eine signifikant niedrigere Ausprägung in sozialer Einbindung. Auch der sozioökonomische Status stellte sich als signifikanter Prädiktor für die soziale Einbindung heraus (F = 21,75, *p* < 0,001). So waren sowohl ein mittlerer (β = 0,64, *p* = 0,008) als auch ein hoher sozioökonomischer Status (β = 1,57, *p* < 0,001) mit einer signifikant höheren sozialen Einbindung assoziiert als ein niedriger. Eine ausgeprägtere depressive Symptomatik ging signifikant mit einer geringeren sozialen Einbindung einher (β = −0,18, *p* < 0,001). Für den BMI zeigte sich kein signifikanter Zusammenhang zum Grad der sozialen Einbindung (β = −0,01, *p* = 0,700).

**Tab. 4:** Assoziierte Faktoren von sozialer Einbindung – Ergebnisse der linearen Regressionsanalyse^a^ (*N* = 8350)

	β	95 %-KI	Wald/F	*p*-Wert
**Soziodemografische Charakteristika**				
*Alter*	−0,11	−0,12; −0,09	–	**< 0,001**
*Geschlecht (Männlich* *=* *Ref.)*				
Weiblich	0,81	0,49; 1,13	–	**< 0,001**
*Familienstand (verheiratet, zusammenlebend* *=* *Ref.)*	–	–	20,77	**< 0,001**
Verheiratet, getrennt lebend	−1,40	−2,07; −0,73	–	**< 0,001**
Ledig	−1,36	−1,83; −0,90	–	**< 0,001**
Geschieden	−1,63	−2,03; −1,24	–	**< 0,001**
Verwitwet	−0,18	−0,70; 0,34	–	0,497
**Sozioökonomische Charakteristika**				
*Sozioökonomischer Status (niedrig* *=* *Ref.)*	–	–	21,75	**< 0,001**
Mittel	0,64	0,17; 1,12	–	**0,008**
Hoch	1,57	1,05; 2,10	–	**< 0,001**
**Gesundheitsbezogene Charakteristika**				
*Depressive Symptomatik*^*b*^	−0,18	−0,21; −0,16	–	**< 0,001**
*BMI*	−0,01	−0,04; 0,03	–	0,700

Anmerkungen. Ergebnisse gewichtet nach Alter und Geschlecht gemäß den Zensus-Daten 2011; fett markierte *p*-Werte sind signifikant

*BMI* Body-Mass-Index, *Ref.* Referenzgruppe, *F* F-Statistik, *β* Regressionskoeffizient, *KI* Konfidenzintervall, *N* Stichprobengröße

^a^Höhere Werte des LSNS-6-Scores werden im Sinne einer höheren sozialen Einbindung interpretiert

^b^Gemessen mit der Allgemeinen Depressionsskala (ADS)

## Diskussion

Ziel der vorliegenden Studie war es, insbesondere die Prävalenz sozialer Isolation bei Menschen mit und ohne Adipositas für eine urbane Erwachsenenpopulation im Alter von 18–79 Jahren zu untersuchen. Es zeigte sich, dass ein substantieller Anteil von Studienteilnehmenden von sozialer Isolation betroffen war (13,1 %). Menschen mit Adipositas zeigten eine nahezu doppelt so hohe Prävalenz sozialer Isolation (20,4 %) gegenüber Personen ohne Adipositas (11,4 %). Damit wurden erstmals anhand einer populationsbasierten Stichprobe unter Einbezug von jüngeren sowie älteren Erwachsenen signifikante Prävalenzunterschiede für soziale Isolation bei Menschen mit und ohne Adipositas für Deutschland nachgewiesen. Bisherige Studien zum Zusammenhang von Adipositas und sozialer Isolation sind rar und lieferten heterogene Befunde [35], zudem inkludierten sie ausschließlich Bevölkerungsgruppen des zumeist höheren Erwachsenenalters. Jüngere Erwachsene wurden bisher nicht in Untersuchungen eingeschlossen. In einer Studie mit Hochaltrigen (85+ Jahre) von Hajek et al. [35] konnte im Gegensatz zur vorliegenden Studie kein Zusammenhang zwischen Adipositas und sozialer Isolation gefunden werden. In einer anderen Studie zeigte sich hingegen, dass Adipositas bei Frauen über 40 Jahren mit einer geringeren sozialen Isolation einherging [36]. Im Weiteren beobachteten Hajek et al. [37] in einer repräsentativen Studie einen Rückgang des Isolationserlebens bei Gewichtsverlust für Frauen ab 40 Jahren. Für Männer (40+ Jahre) zeigte sich derweilen in den beiden zuvor genannten Studien kein Zusammenhang. Fehlende oder zunächst kontraintuitiv erscheinende Zusammenhänge von Adipositas und sozialer Isolation könnten durch Faktoren wie Geselligkeit oder eine positivere Einstellung gegenüber Adipositas im höheren Lebensalter bedingt sein [35, 38]. Unterschiede in den Studienergebnissen könnten auch auf unterschiedliche Instrumente zur Messung sozialer Isolation zurückzuführen sein.

Während ein höherer BMI in der vorliegenden Untersuchung nicht per se eine schlechtere soziale Einbindung prädizierte, zeigte sich jedoch, dass sozial isolierte Menschen mit Adipositas im Vergleich zu jenen ohne Adipositas eine besondere Risikogruppe für eine eingeschränkte psychische Gesundheit darstellen. Über Faktoren, die ursächlich für eine erhöhte Prävalenz sozialer Isolation und ein verringertes soziales Netzwerk bei Menschen mit Adipositas sind, können lediglich Vermutungen angestellt werden. Einschränkungen in der Mobilität können die Aufrechterhaltung von Sozialkontakten erschweren [11] und soziale Aktivität verringern [39]. Zudem können die psychosozialen Auswirkungen von Adipositas zur Erklärung des Zusammenhanges beitragen. Einerseits könnte eine allgegenwärtige gesellschaftliche Stigmatisierung und Diskriminierung eine soziale Isolation von Menschen mit Adipositas fördern [13]. Andererseits könnte auch die Internalisierung negativer gewichtsbezogener Vorurteile verbunden mit Gefühlen von Selbsthass, Selbstabwertung und Inkompetenz [40, 41] einen sozialen Rückzug betroffener Personengruppen begünstigen.

Menec et al. [42] heben hervor, dass es zur Entwicklung und Verfeinerung von Präventions- und Interventionsmaßnahmen nützlich ist, Einsamkeit und soziale Isolation gemeinsam zu betrachten. Eine gemeinsame Erfassung beider Konstrukte sollte daher in zukünftigen Studien Berücksichtigung finden. Während soziale Isolation häufig als objektives Maß einen geringen Umfang des sozialen Netzwerkes anzeigt, spiegelt Einsamkeit die gefühlte Diskrepanz zwischen tatsächlichem und gewünschtem In-Gesellschaft-Sein wider [43]. So können sich Personen trotz objektiv geringer quantitativer und qualitativer sozialer Kontakte nicht einsam fühlen und andere wiederum trotz starker sozialer Einbettung Einsamkeit empfinden. In einer systematischen Übersichtsarbeit von Lam et al. [44] über die Neurobiologie der Einsamkeit wurde festgestellt, dass Einsamkeit mit bestimmten Hirnregionen und -netzwerken in Verbindung steht (z. B. dem präfrontalen Cortex, der Insula, der Amygdala, dem Hippocampus). In einer weiteren Metaanalyse von Park et al. [45] weisen die Autoren darauf hin, dass chronische Einsamkeit zu chronischen Stressreaktionen führen kann, welche nachgelagerte Entzündungsreaktionen sowie negatives Gesundheitsverhalten bei den Betroffenen auslösen können, welches schließlich in negativen gesundheitlichen Folgen resultiert. Diese Prozesse werden als komplex beschrieben und funktionieren bidirektional [45]. Die Untersuchung relevanter, auch neurobiologischer Wirkmechanismen sollte daher Gegenstand zukünftiger Forschungsarbeiten sein. Insgesamt bleibt festzuhalten, dass Menschen mit Adipositas auf Basis des vorliegenden Studienergebnisses eine mögliche Risikogruppe für das Erleben sozialer Isolation darstellen.

In Anbetracht der weitreichenden negativen gesundheitlichen Folgen von sozialer Isolation und Adipositas [4, 46] wurde explorativ den Gruppenunterschieden hinsichtlich soziodemografischer und sozioökonomischer Faktoren zwischen sozial Isolierten mit und ohne Adipositas nachgegangen. Sozial Isolierte mit Adipositas wiesen im Mittel ein signifikant höheres Lebensalter auf. Mit zunehmendem Alter steigen gleichermaßen das Adipositasrisiko [5, 47] und das Risiko sozialer Isolation [48], weshalb auch das Durchschnittsalter sozial isolierter Personen mit komorbider Adipositas höher ausfallen könnte. So zeigte sich auch in der vorliegenden Studie, dass höheres Alter im Rahmen des Regressionsmodells signifikant mit einem geringeren Grad der sozialen Einbindung einherging. Die mögliche Interaktion zwischen Alter und BMI auf die soziale Einbindung wurde statistisch geprüft, erwies sich jedoch als nicht signifikant. Andere Studien weisen dagegen auf Alter als möglichen Confounder aufgrund des Alterungsprozesses und Veränderungen in der Körperfettverteilung hin [49, 50].

Des Weiteren zeigte sich, dass eine bessere soziale Einbindung signifikant mit weiblichem Geschlecht, einem verheirateten (und zusammenlebenden) Familienstand sowie einem höheren sozioökonomischen Status einherging. Demgegenüber ließen sich jedoch keine statistisch signifikanten Unterschiede zwischen sozial Isolierten mit und ohne Adipositas in diesen Variablen finden. Eine geringere soziale Einbindung war in der vorliegenden Untersuchung signifikant mit einer höheren depressiven Symptomatik assoziiert. Im Gruppenvergleich zeigte sich dabei, dass sozial isolierte Menschen mit Adipositas signifikant stärker von Depressivität betroffen sind als sozial Isolierte ohne Adipositas. Die mögliche Interaktion zwischen Depressivität und BMI auf die soziale Einbindung wurde statistisch geprüft, erwies sich jedoch als nicht signifikant. Bisherige Studien zeigten, dass Adipositas und soziale Isolation unabhängig voneinander Risikofaktoren für das Auftreten depressiver Symptome sind [14, 17, 22]. Sozial isolierte Personen mit Adipositas könnten einer doppelten Belastung ausgesetzt sein. Einerseits müssen sie mit dem psychosozialen Stress der sozialen Isolation umgehen und andererseits sehen sie sich mit den physischen und psychischen Herausforderungen der Adipositaserkrankung konfrontiert. Zudem ist von komplexen Wechselwirkungen zwischen den Faktoren auszugehen. Beispielsweise kann umgekehrt eine Depression ein soziales Rückzugsverhalten [51] und gleichermaßen die Entwicklung einer Adipositas fördern [21, 22]. Hierbei werden eine größere physische Inaktivität und ungesündere Essenspräferenzen als Gründe diskutiert [22, 52]. So ist denkbar, dass Menschen, die von sozialer Isolation betroffen sind, ihren erlebten Stress über dysfunktionale Bewältigungsstrategien, wie z. B. Emotionsregulation über die Nahrungsaufnahme, regulieren [53]. Eine Gewichtszunahme kann ebenso durch die Einnahme von Antidepressiva [54] oder durch eine eingeschränkte Schlafqualität bedingt sein [55, 56]. Auch neuroendokrine Prozesse, wie eine erhöhte Aktivität der HPA-Achse bei Menschen mit einer Depression, werden als mögliche Einflussfaktoren bei der Entstehung von Adipositas diskutiert [57, 58].

Insgesamt lässt sich schlussfolgern, dass es sich bei sozial isolierten Personen mit einer komorbiden Adipositas um eine besondere Risikogruppe hinsichtlich einer gefährdeten psychischen Gesundheit handelt. Insbesondere vor dem Hintergrund einer erhöhten Prävalenz sozialer Isolation bei Adipositas sollte dieser Risikogruppe innerhalb der klinischen Versorgung eine höhere Aufmerksamkeit geschenkt werden. Um sozialer Isolation und Einsamkeit bei Menschen mit Adipositas entgegenzuwirken, gilt es, sowohl aufseiten der Behandlerinnen und Behandler als auch in der Bevölkerung insgesamt nichtstigmatisierende Grundhaltungen zu fördern [59]. Hierzu gehört auch, die Vielfalt menschlicher Körper zu normalisieren und die Öffentlichkeit besser aufzuklären [60]. Beispiele dafür sind die *Fat-Acceptance*- oder *Body-Positivity-Bewegung* und der *Health-at-Every-Size-Ansatz*, welche u. a. die Entwicklung von Selbstliebe und Selbstbewusstsein von Menschen mit Übergewicht fördern und gleichzeitig ein positives Bild in der Öffentlichkeit durch z. B. soziale Medien aufbauen [61]. Es geht in diesen Ansätzen darum, für Menschen mit Adipositas geschützte Austauschmöglichkeiten zu schaffen, um sich verbinden zu können und die soziale Einbindung zu steigern. Darüber hinaus braucht es auch auf politischer und gesellschaftlicher Ebene mehr Aufmerksamkeit für Menschen mit Adipositas, um Benachteiligungen und Ausgrenzungen aufgrund des Körpergewichts zu reduzieren [61]. Dies könnte u. a. durch Awareness-Kampagnen, Veranstaltungen und Workshops mit den Themen Gewichtsdiskriminierung und -stigmatisierung umgesetzt werden.

### Stärken und Limitationen

Eine Stärke der vorliegenden Studie ist die umfassende populationsbasierte Stichprobe, anhand derer die Forschungsfragen untersucht wurden. Die Daten der LIFE-Adult-Studie ermöglichen unter Anwendung eines Gewichtungsfaktors repräsentative Ergebnisse für die deutsche Erwachsenenbevölkerung. Trotz genannter Stärken bestehen auch einige Einschränkungen. Die Forschungsfragen wurden im Querschnittsdesign untersucht, weshalb lediglich korrelative und keine kausalen Aussagen getroffen werden können. Des Weiteren basieren die verwendeten Instrumente, den BMI ausgenommen, überwiegend auf Selbstauskünften. Obwohl zur Bewertung von sozialer Isolation und einer depressiven Symptomatik mit der ADS und der LSNS‑6 validierte und häufig eingesetzte Instrumente herangezogen wurden, basieren diese auf Selbstberichten der Studienteilnehmenden und können daher anfällig für eine Verzerrung sein. Im Weiteren wurde anhand einer Missing-Analyse deutlich, dass verschiedene soziodemografische und sozioökonomische Variablen, wie z. B. ein höheres Alter, ein verwitweter Familienstand, männliches Geschlecht oder ein geringerer SES, das Auftreten fehlender Werte in der Baseline-Stichprobe wahrscheinlicher machten, was möglicherweise zu einer Verzerrung der Ergebnisse geführt haben könnte.

## Fazit

Zusammenfassend gibt die Arbeit Einblicke in die komplexen Zusammenhänge von Adipositas, sozialer Isolation und der psychischen Gesundheit für die deutsche Erwachsenenbevölkerung. Dabei werden erstmals auch Daten für Erwachsene jüngerer Altersgruppen mit einbezogen. Weitere längsschnittliche Forschung ist notwendig, um die Assoziationen und Wechselwirkungen zwischen den Faktoren besser zu verstehen. Es gilt, passgenaue Präventions- und Unterstützungsmaßnahmen für diese Zielgruppe zu entwickeln, um psychischen Erkrankungen vorzubeugen bzw. die mentale Gesundheit von Betroffenen zu verbessern. Interdisziplinären Versorgungsangeboten kommt hier eine besondere Bedeutung zu [62].
